# The structure of F_1_-ATPase from *Saccharomyces cerevisiae* inhibited by its regulatory protein IF_1_

**DOI:** 10.1098/rsob.120164

**Published:** 2013-02

**Authors:** Graham C. Robinson, John V. Bason, Martin G. Montgomery, Ian M. Fearnley, David M. Mueller, Andrew G. W. Leslie, John E. Walker

**Affiliations:** 1 The Medical Research Council Mitochondrial Biology Unit, Hills Road, Cambridge CB2 0XY, UK; 2 Rosalind Franklin University of Medicine and Science, The Chicago Medical School, North Chicago, IL 60064, USA; 3 The Medical Research Council Laboratory of Molecular Biology, Hills Road, Cambridge CB2 0QH, UK

**Keywords:** F_1_-ATPase, natural inhibitor, catalysis, intermediate

## Abstract

The structure of F_1_-ATPase from *Saccharomyces cerevisiae* inhibited by the yeast IF_1_ has been determined at 2.5 Å resolution. The inhibitory region of IF_1_ from residues 1 to 36 is entrapped between the C-terminal domains of the α_DP_- and β_DP_-subunits in one of the three catalytic interfaces of the enzyme. Although the structure of the inhibited complex is similar to that of the bovine-inhibited complex, there are significant differences between the structures of the inhibitors and their detailed interactions with F_1_-ATPase. However, the most significant difference is in the nucleotide occupancy of the catalytic β_E_-subunits. The nucleotide binding site in β_E_-subunit in the yeast complex contains an ADP molecule without an accompanying magnesium ion, whereas it is unoccupied in the bovine complex. Thus, the structure provides further evidence of sequential product release, with the phosphate and the magnesium ion released before the ADP molecule.

## 2. Introduction

The hydrolysis of ATP by the F-ATPase isolated from mitochondria is inhibited by a small basic protein, known as IF_1_ [1]. When the purified enzyme was co-reconstituted into phospholipid vesicles with bacteriorhodopsin, IF_1_ had no effect on ATP synthesis, whereas ATP hydrolysis by the purified enzyme was inhibited [2]. The binding of IF_1_ to the enzyme requires the hydrolysis of ATP to drive the anticlockwise rotation of the central stalk (as viewed from the membrane domain of the intact enzyme) and entrap the inhibitor in its binding site. Bovine IF_1_, the most extensively characterized ATPase inhibitor protein, consists of a chain of 84 amino acids [3,4]. Residues 21–83 are folded into a long α-helix, and the active form is a dimer held together by an antiparallel coiled-coil of α-helices from residues 49 to 81 [5–7]. The inhibitory region is found in residues 1–46 [8], and the dimeric inhibitor can inhibit two F_1_-ATPase complexes simultaneously [9]. A monomeric form, prepared by deleting residues 61–84, is also an effective inhibitor. The structure of bovine F_1_-ATPase inhibited with this monomeric inhibitor (known as F_1_-I1–60) shows the N-terminal region of IF_1_ from residues 1–13 lying within the aqueous cavity surrounding an α-helical coiled-coil in the γ-subunit that forms the rotor of F_1_-ATPase; residues 1–7 were unresolved and residues 8–13 form an extended structure [8]. Residues 14–18 are folded into a single turn of an α-helix, which interacts with the coiled-coil region of the γ-subunit; residues 19 and 20 link this short α-helix to the long α-helix of the inhibitor, which extends outwards from residue 21 beyond the external surface of the α_3_β_3_-domain of the enzyme. Residues 21–46 of this long α-helix occupy a deep groove, formed mainly by α-helices and loops in the C-terminal domains of the α_DP_- and β_DP_-subunits in a catalytic interface of F_1_-ATPase, and hydrophobic interactions between the inhibitor and the β_DP_-subunit provide most of the binding energy [10].

**Figure 1.**
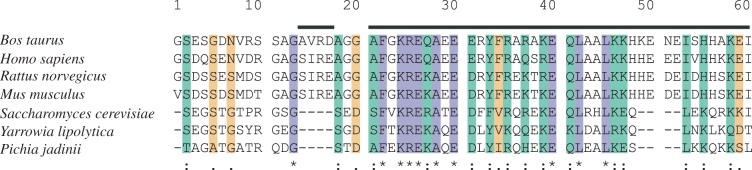
Alignment of the sequences of residues 1–60 of bovine IF_1_, and the equivalent region of yeast IF_1_, with the same regions from other species. The purple, green and yellow stripes denote identical, highly conserved and poorly conserved residues, respectively. The alignment was performed with ClustalW. The bars above the sequences denote α-helical regions in the bovine protein. The yIF_1_ used in crystallization experiments contained the mutation E21A.

The ATPase inhibitor protein from *Saccharomyces cerevisiae* is 63 amino acids long. The poorly conserved N-terminal region (residues 1–16; figure 1), is followed by a well-conserved segment from residues 17 to 45, corresponding to the long α-helix in the inhibitory region of the bovine protein. However, the C-terminal segment responsible for the formation of dimers in the bovine protein is truncated and not conserved in the yeast protein.

As described here, the structure of yeast F_1_-ATPase inhibited with residues 1–53 of yeast IF_1_ (yI1–53) has been determined at 2.5 Å resolution. Many features of this structure are similar to those of the structure of bovine F_1_-I1–60. However, one significant difference is that the yeast inhibitor has arrested the catalytic cycle of ATP binding and hydrolysis followed by product release at an earlier stage in the cycle than the bovine inhibitor. This structure provides independent confirmation of a new intermediate in the catalytic cycle of F_1_-ATPase, observed in a structure of bovine F_1_-ATPase [11], which immediately precedes the formation of the ‘open’ or ‘empty’ state observed in the ‘ground state’ structure.

## 3. Results

### 3.1. Oligomeric states of inhibitor proteins

**Figure 2.**
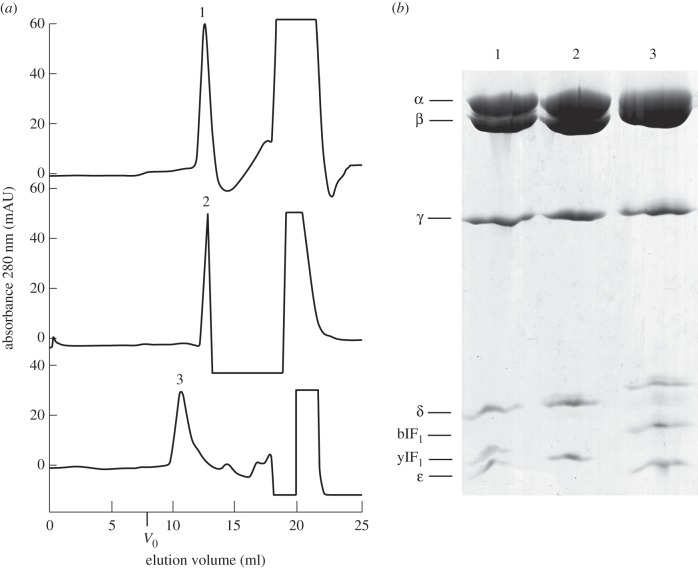
Gel filtration chromatography of yeast and bovine F_1_-ATPase-IF_1_ complexes. The yeast and bovine enzymes were inhibited with the inhibitor protein from *S. cerevisiae* (yF_1_) and with bovine IF_1_ (bIF_1_), respectively. (*a*) Column profiles of yF_1_–yIF_1_, active yF_1_ and bF_1_–bIF_1_ complexes, respectively. *V*_0_ is the void volume of the column. (*b*) SDS–PAGE analysis of peaks 1, 2 and 3 from (*a*).

The complex of yeast F_1_-ATPase inhibited with full-length yeast IF_1_ was estimated by gel filtration chromatography to have an apparent molecular mass of 385 kDa, whereas the value for the complex between the bovine F_1_-ATPase and full-length bovine IF_1_ was 670 kDa (figure 2). These data are consistent with the yeast and bovine F_1_-IF_1_ complexes being monomeric and dimeric, respectively, with the dimeric bovine inhibitor bound to two F_1_-ATPase complexes, as demonstrated before [6].

### 3.2. Structure determination

**Figure 3.**
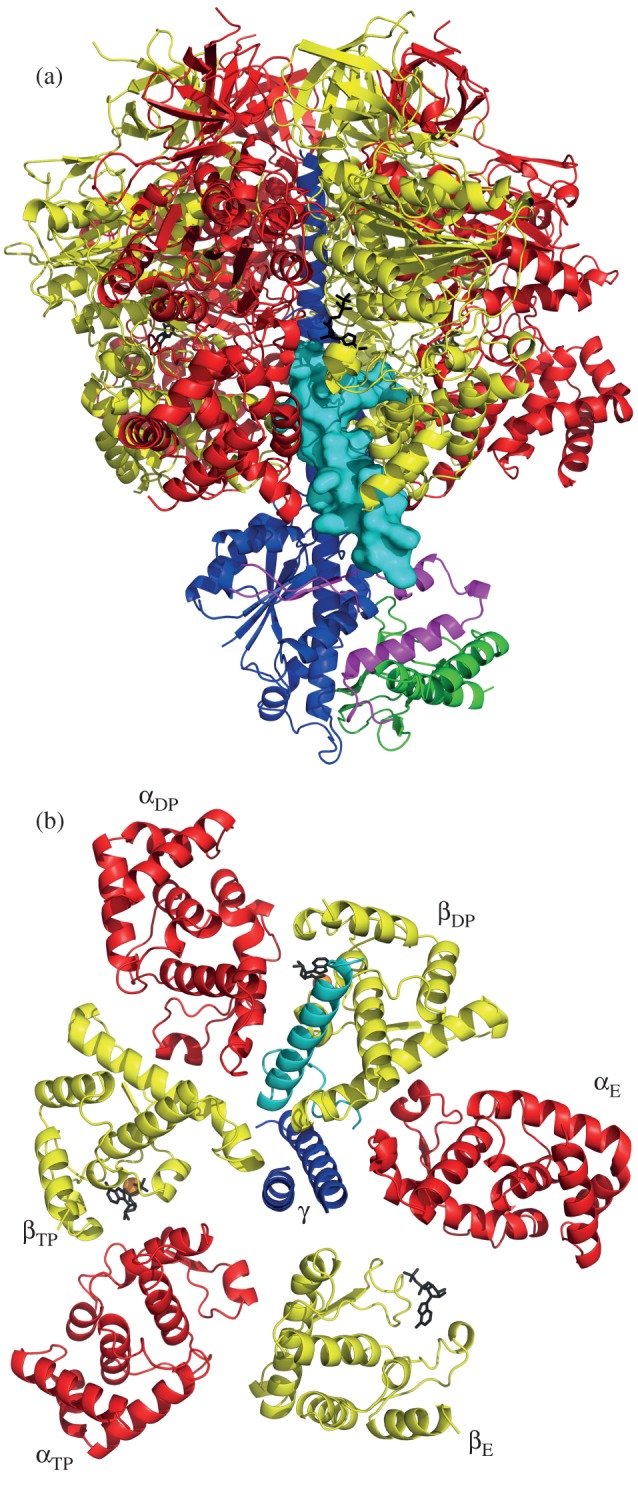
The structure of the F_1_-I1–53 complex from *S. cerevisiae*. The α-, β-, γ-, δ- and ε-subunits are depicted in ribbon form in red, yellow, dark blue, green and magenta, respectively, and residues 1–36 of I1–53 are light blue. (*a*) Overall view of the complex viewed from the side with IF_1_ shown in solid representation. (*b*) View (upwards from the foot of the central stalk) along the axis of the γ-subunit showing the position of the α-helix of yeast IF_1_ in ribbon representation relative to the C-terminal domains of α- and β-subunits.

The inhibited complex between yeast F_1_-ATPase and yeast I1–53, known as yF_1_-I1–53, was formed in the presence of Mg-ATP as described in §5. Its structure (figure 3) was determined by X-ray crystallography, and was solved by molecular replacement (see §5) with data at 2.5 Å resolution. The asymmetric unit contains two yF_1_-I1–53 complexes. Data processing and refinement statistics are summarized in table 1. The final model of yF_1_-I1–53 contains the following residues: α_E_, 26–509; α_TP_, 25–406 and 412–509; α_DP_, 26–509; β_E_, 8–475; β_TP_, 7–475; β_DP_, 6–475; γ, 1–59 and 71–276; δ, 11–23 and 27–137; ε, 1–49 and 53–61 and yI1–53, 1–36. The refined temperature factors suggested the presence of a mixture of Mg-ATP (75%) and Mg-ADP (25%) in the nucleotide binding site of the non-catalytic α_E_-subunit, whereas the nucleotide binding sites in the α_TP_-, β_DP_- and β_TP_-subunits all contained Mg-ADP only. The nucleotide binding site of the α_DP_-subunit was occupied almost entirely by Mg-ADP, but there was also evidence for the presence of Mg-ATP at low occupancy. The β_E_-subunit contained a bound ADP molecule only, and there was no electron density corresponding to either bound phosphate or a magnesium ion. The models of both assemblies in the asymmetric unit contain the same residues and have the same nucleotide occupancy. Their structures are essentially identical (the r.m.s. value from a global superimposition was 0.34 Å; the values for individual subunits are between 0.01 and 0.02 Å).

**Table 1.** Data collection and refinement statistics for the complex between yeast F_1_-ATPase and the yeast inhibitor protein, yI1–53. Statistics for the highest resolution bin (2.64–2.5 Å) are shown in parentheses.

space group	P2_1_
unit cell dimensions *a, b, c* (Å); β (^o^)	118.2, 187.8, 181.8; 90.0
resolution range, Å	43.84–2.5 (2.64–2.5)
no. unique reflections	268 620 (38 863)
multiplicity	3.9 (3.9)
completeness, %	98.4 (97.6)
*R*_merge_^a^	10.9 (74.8)
*〈I/*σ**(*I*)〉	8.7 (2.0)
*B* factor, from Wilson plot, Å^2^	56.0
water molecules	735
*R* factor^b^	22.44%
free *R* factor^c^	26.19%
r.m.s. of bonds, Å	0.009
r.m.s. of angles, °	1.2

^a^*R*_merge_ = ∑_h_∑*_i_*|*I*(*h*) − *I*(*h*)_i_|∑_h_∑_i_*I*(*h*)*_i_*, where *I*(*h*) is the mean weighted intensity after rejection of outliers.

^b^*R* factor = ∑_hlk_||*F*_obs_| − *k*|*F*_calc_||/∑_hlk_|*F*_obs_|, where *F*_obs_ and *F*_calc_ are the observed and calculated structure factor amplitudes, respectively.

^c^*R*_free_ = ∑_hkl⊂_*_T_*||*F*_obs_| − *k*|*F*_calc_||/∑_hkl⊂T_||*F*_obs_|, where *F*_obs_ and *F*_calc_ are the observed and calculated structure factor amplitudes, respectively, and *T* is the test set of data omitted from refinement (5% in this case).

### 3.3. Structure of the yeast F_1_-IF_1_ complex

**Figure 4.**
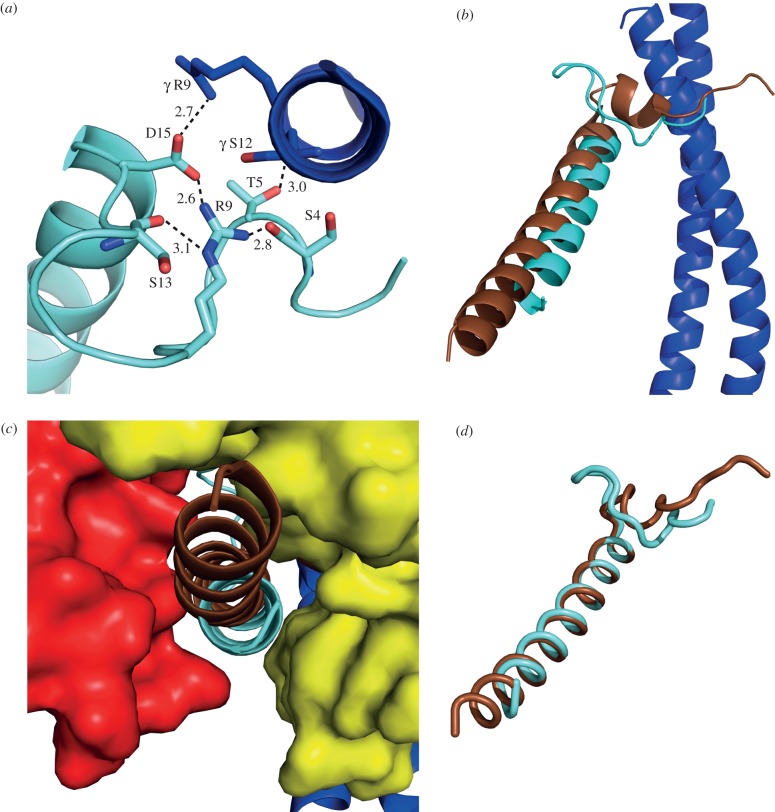
The binding site for yeast I1–53 in the structure of yeast F_1_-ATPase. (*a*) The N-terminal loop region of the inhibitor protein (light blue) in juxtaposition with the C-terminal helix of the γ-subunit (dark blue). Dotted lines represent possible interactions with distances in angstrom. (*b*) View from the side of the central stalk showing the orientations of the yeast and bovine inhibitor proteins (light blue and brown, respectively) relative to the central stalk. (*c*) View from outside the F_1_-domain towards the γ-subunit of the enzyme (dark blue) of the deep cleft between the C-terminal domains of the α_DP_- and β_DP_-subunits (red and yellow, respectively) where the α-helical region of I1–53 (light blue) is bound. The position of the equivalent region of the bovine inhibitor protein in the structure of bovine F_1_-I1–60 is shown in brown. (*d*) View of the bovine inhibitor (brown) superposed onto the yeast inhibitor protein (light blue) via residues 22–25 and 17–20 in the bovine and yeast inhibitors, respectively.

In the structure (figure 3), the resolved part of yeast I1–53 is found between the C-terminal domains of the α_DP_- and β_DP_-subunits. This region consists of an extended structure from residues 1 to 16, bent into a loop from residues 6 to 16 (figure 4*a*), with residues 17–36 folded into an α-helix 29 Å long (figure 4*b*). Residues 17–35 of the α-helix are bound into a deep groove between the C-terminal domains of the α_DP_- and β_DP_-subunits (figure 4*c*). The polypeptide chain beyond residue 36 presumably extends from the external surface of the F_1_-domain. Residues 1–5 of the inhibitor are in the aqueous chamber surrounding the γ-subunit in the central region of the F_1_-domain. Residue E2 makes a salt bridge with residue α_E_-K361 in α-helix H (see Abrahams *et al*. [12] for definition of secondary structure elements) in the nucleotide binding region of the α_E_-subunit. Residues 6–16 form a loop region held together by a salt bridge between residues R9 and D15 of the inhibitor, and by a hydrogen-bonding network involving residues S4, R9 and D15 of the inhibitor (figure 4*a*). In this region, the structure of the yeast inhibitor differs from that of the N-terminal region of the bovine inhibitor bound to bovine F_1_-ATPase (figure 4*b*), reflecting the difference in length between the yeast and bovine inhibitors and the lack of sequence similarity in their N-terminal regions (figure 1). Nonetheless, the two N-terminal regions occupy a similar space in the respective F_1_-ATPases (figure 4*b*). The loop motif in residues 6–16 of the yeast inhibitor protein replaces the motif consisting of the α-helical turn and the following extended region in the bovine protein. However, both motifs are in contact with the N-terminal α-helix of the γ-subunit. From residue 17, the yeast inhibitor forms an α-helix that extends to residue 36 in the structure. The bovine protein contains a similar α-helix beginning at residue 21, one residue before the yeast protein in the aligned sequences and structures, and continuing up to residue 50. These α-helical regions of the bovine and yeast inhibitor proteins are bound in a related way, and occupy the same cleft between the C-terminal domains of the α_DP_- and β_DP_-subunits. Most of the cleft lies between the C-terminal ends of helices 1 and 2 in the C-terminal domains of the β_DP_- and α_DP_-subunits. The entrance to the cleft from the central cavity is close to the C-terminal end of helix 1 in the C-terminal domain of the β_TP_-subunit, and the exit to the exterior surface of the F_1_-domain is completed by loops between helices 1 and 2 in the C-terminal domain of the α_DP_-subunit, and between helices 4 and 5 in the C-terminal domain of the β_DP_-subunit.

Although there are similarities between the binding modes of the α-helices of the yeast and bovine inhibitor proteins, they are not identical. Global superimposition via the Cα-atoms of the two inhibited structures demonstrates their overall similarity (figure 4*b*; r.m.s. value 2.48 Å), and the structures of the inhibitors themselves in the inhibited complexes are also similar (figure 4*d*). However, in the inhibited complexes, the α-helices of yeast I1–53 and bovine IF_1_ do not lie exactly on the same axis; the α-helix of the yeast protein follows a steeper path, relative to the approximately vertical central stalk of F_1_-ATPase, and the paths of the bound inhibitors diverge increasingly towards the outside the F_1_-domain with an angle of *ca* 7° between the α-helices. The most obvious reason for the slightly different binding position of IF_1_ in the bovine and yeast enzymes is a significant alteration in the conformation of residues 391–398 of the β_DP_-subunit of F_1_-ATPase; for example, the positions of the Cα atoms of residues 392 and 393 differ by 1.6 and 2.7 Å, respectively. Residues 391–398 of the β_DP_-subunit help to form the ‘base’ of the binding pocket for IF_1_, and the displacement of this region in the yeast enzyme relative to the bovine enzyme accompanies the downward displacement of the long α-helix of IF_1_. In both the bovine and yeast F_1_-IF_1_ structures, residues 382–398 of the β_DP_-subunit are the region that deviates most from the bovine ground state structure. Its change in conformation is associated with the binding of IF_1_, and it is reasonable to suggest that this difference between the bovine and yeast F_1_-IF_1_ structures reflects how each enzyme adapts in order to bind the different sequences of bovine and yeast IF_1_, resulting in the slightly different binding modes. These slightly differing binding modes are illustrated by the superimposed structures of the yeast and bovine inhibitor proteins in the F_1_-IF_1_ complexes (figure 4*b*,*c*). One specific interaction supports this interpretation. In the bovine complex, β_DP_-D394 interacts with R32 of IF_1_ to form a salt bridge, and the position of the loop containing residues 391–398 is influenced and displaced by γR133. In the yeast enzyme, βD394 is conserved, but the bovine IF_1_ residue R32 is replaced by F27 in yeast IF_1_, and the different conformation of yeast β_DP_-residues 391–398 arises from β_DP_-D394 moving away from the hydrophobic side chain of yeast inhibitor residue F27, and the position of the loop is no longer influenced by γK135, the equivalent of bovine γR133 (see the electronic supplementary material, figure S1).

In consequence of these slightly different modes of binding, there are both similarities and differences in the detailed interactions between the inhibitor proteins and their cognate F_1_-ATPases. The residues in the long α-helix of bovine IF_1_ that contribute significantly to its binding to bovine F_1_-ATPase have been identified by mutagenesis of each residue in the α-helix, and by quantitative measurement of the impact of each mutation on binding [10]. These experiments have shown that the bovine α-helix is bound mainly by hydrophobic interactions between residues Y33, F34, Q41, L42 and L45 of the inhibitor protein and hydrophobic side chains in the C-terminal domain of the β_DP_-subunit, and F22 with the C-terminal domain of the β_TP_-subunit. In addition, bovine inhibitor residue Q41 contributes by making polar interactions with the same region of the β_DP_-subunit, and there is a salt bridge between inhibitor residue E30 and residue R408 in the β_DP_-subunit.

The structure of yF_1_-I1–53 shows that residues F17, E25 and F28 in yI1–53 and the equivalent residues, F22, E30 and Y33, in the bovine inhibitor, interact with their cognate F_1_-ATPases in the same manner. However, the other interactions noted in the bovine complex are not conserved, but the structure indicates that there are additional significant interactions that are specific to the yeast complex. They are a salt bridge between residue E2 of the inhibitor with residue K361 in α-helix H of the α_E_-subunit (figure 4*c*), and between R32 of the inhibitor and E398 and E405 in the β_DP_-subunit, and an electrostatic interaction between inhibitor residue D15 and R9 in the γ-subunit. In addition, inhibitor residues D15 and R9 form an ionic interaction, evidently helping to stabilize the loop from inhibitor residues 6–16 (figure 4*a*), and E33 makes a backbone interaction with the amide nitrogen of β_DP_-E454. There are other yeast specific interactions between inhibitor residues T5, G6 and S11 with γS12, β_DP_D386 and β_DP_E341, respectively.

**Figure 5.**
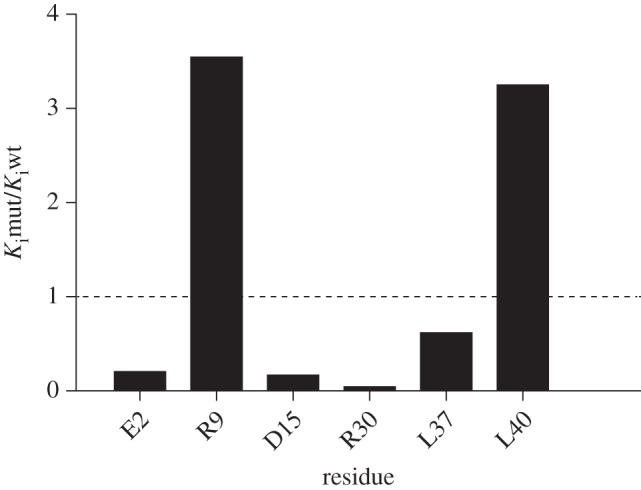
Influence of point mutation of selected residues in yI1–53 on its inhibition of yeast F_1_-ATPase. The quantitative data from which the figure is derived are given in table 2. *K*_i_wt and *K*_i_mut are the dissociation constants for the wild-type and mutant proteins, respectively.

In order to investigate some of these detailed differences between the mode of binding of the yeast and bovine inhibitors, residues E2, R9 and D15 of yeast IF_1_ were substituted singly by alanine residues, as was residue R30, which has been proposed to be important for forming the inhibited complex [13]. In addition, two conserved leucine residues, L37 and L40, were mutated to alanine. The corresponding residues L42 and L45 interact with F_1_-ATPase in the bovine-inhibited complex, but no equivalent interaction is found in yF_1_-I1–53. The results of the quantitative study of the binding properties of each of these mutated proteins are presented in table 2, and the effects of the mutations are summarized in figure 5 as *K*_i_mut/*K*_i_wt, the quotient of the dissociation constants of the mutant and wild-type proteins. Values of the quotient that are greater than and less than unity correspond to proteins with decreased and increased binding to F_1_-ATPase, respectively. The experiments show that among the three N-terminal residues E2, R9 and D15 that were mutated, only the mutation of R9A had a significant effect in increasing the quotient. However, because the substitution D15A had little effect on the quotient (although it changed the dynamics of binding significantly), the importance of R9 in the formation of the inhibited complex does not involve the formation of a salt bridge with residue D15. In the absence of detailed structures of the mutated forms bound to F_1_-ATPase, the impact of these mutations is difficult to assess. The mutation L40A leads to an increase in the quotient, indicating that this residue may have a role at some unidentified intermediate state in the pathway of the formation of the final inhibited complex, as has been suggested for other residues in the bovine complex [10]. The substitution L37A had little impact on the quotient, and the reason for its conservation remains obscure. Finally, the mutation R30A, rather than abolishing the inhibitory activity of yIF_1_, as reported [13], decreased the quotient slightly.

**Table 2.** The binding and dissociation rate constants for yI1–53His proteins containing point mutations.

mutation	*k*_on_ × 10^−2^ (μM^−1^ s^−1^)	*k*_off_ × 10^−2^ (s^−1^)	*K*_i_ × 10^−2^ (μM^−1^)
none	58.3 ± 5.6	2.8 ± 0.3	4.8 ± 0.7
E2A	12.3 ± 0.3	0.1 ± 0.0	1.0 ± 0.1
R9A	11.4 ± 0.8	1.9 ± 0.2	17.0 ± 2.1
D15A	170.7 ± 2.1	1.4 ± 0.6	0.8 ± 0.4
R30A	127.8 ± 6.8	0.3 ± 0.2	0.2 ± 0.2
L37A	19.1 ± 1.0	0.4 ± 0.4	2.2 ± 1.9
L40A	11.6 ± 1.2	1.8 ± 0.3	15.6 ± 2.9

## 4. Discussion

### 4.1. The inhibited state

**Figure 6.**
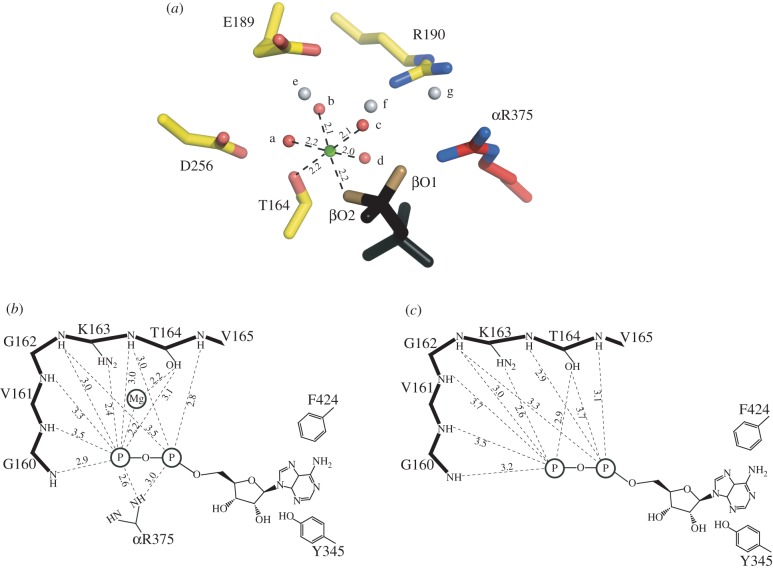
Change in the coordination of a magnesium ion in the active site of yeast F_1_-ATPase. (*a*) Hexacoordination of the magnesium ion (green) in the catalytic site of the β_DP_-subunit. The ligands are provided by water molecule a–d (red), by the oxygen atom βO2 of ADP (or ATP), and by the β-hydroxy-group of β_DP_-T164. The water molecules a–d are themselves hydrogen-bonded to other water molecules e–g (grey), by the oxygen atom βO1 of the nucleotide, and by the side chain functionalities of residues β_DP_-E189, β_DP_-R190 and β_DP_-D256. (*b*) Schematic of the disposition of the magnesium ion and bound ADP in the catalytic site of the β_DP_-subunit. The P-loop sequence (upper left) helps to bind the magnesium ion and the nucleotide. The adenosine moiety of the nucleotide is bound in the hydrophobic pocket between the side chains of residues β_DP_-Y345 and β_DP_-F424. Distances are given in angstrom, and those to the phosphate group are to the closest oxygen atom. (*c*) Rotation of γ-subunit driven by hydrolysis of ATP has opened the nucleotide binding domain of the subunit, converting the β_DP_-site to the β_E_-site. The catalytic ‘arginine finger’ residue, αR375 becomes disordered, and the coordination sphere of the magnesium ion is disrupted, releasing the metal ion. In the structure of yF_1_-I1–53, the inhibitor protein has arrested the conversion of the site just before the formation of a fully ‘empty’ or ‘open’ site. The adenosine binding pocket is still intact, and ADP remains bound to the subunit. In the final step of the conversion of the site to a fully formed β_E_-site (as observed in ‘ground state’ structures of F_1_-ATPase), the side chain of β_E_-F424 rotates through 90°, releasing the ADP molecule.

**Figure 7.**
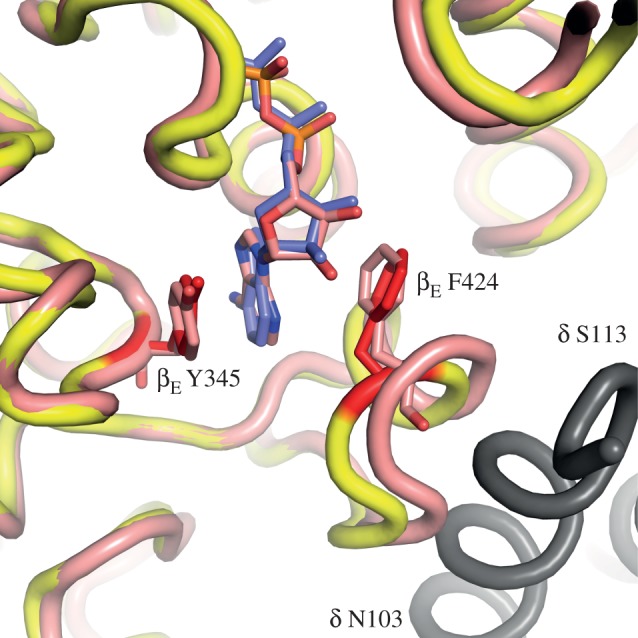
Comparison of the nucleotide binding pockets in the β_E_-subunits in the structures of yF_1_-I1–53 and bovine F_1_-PH. The yeast and bovine protein backbones are coloured yellow and pink, respectively. The side chains of residues β_E_-F424 and β_E_-Y345 are red in the yeast enzyme and pink in the bovine enzyme. They provide the pocket for binding the adenosine moieties of the ADP molecules (blue and pink, respectively in the yeast and bovine enzymes). In grey is shown α-helix b (residues 102–110) in the δ-subunit of an adjacent yeast F_1_-complex in the crystal lattice of yeast F_1_-I1–53. It approaches to within 4 Å of α-helix C3 carrying β_E_-F424 in the yeast structure. Thus, it makes a crystal contact that may influence the position of α-helix C3 in the β_E_-subunit of the yeast enzyme.

One highly significant difference between the structures of the bovine and yeast inhibited complexes is in the occupancy of nucleotides in the β_E_-subunit. In the bovine structure, there is no nucleotide bound to this subunit, whereas in the yeast complex, the still partially formed nucleotide binding site of the β_E_-subunit is occupied by ADP, but without an accompanying bound magnesium ion, despite the presence of 13 mM magnesium sulphate during formation and crystallization of the inhibited complex. In the yeast complex, the C-terminal domains of the α_DP_- and α_TP_-subunits are displaced downwards and outwards, opening the α_DP_–β_DP_ and α_TP_–β_TP_ interfaces slightly relative to the bovine complex. This opening of these two interfaces is accompanied by small, but significant, changes in the β_E_-subunit, where β_E_-Y345 and β_E_-F424 remain sufficiently close to provide a pocket into which the adenosine moiety of ADP can bind (albeit presumably weakly). However, the amino acid side chains that are involved in coordinating a magnesium ion indirectly by binding ligand water molecules (βE189, βE193 and βD256) have moved away from the positions that provide the coordinating environment (as found in the β_DP_- and β_TP_-subunits), and the magnesium ion has been released from the β_E_-subunit (figure 6). Thus, the present structure indicates that the magnesium ion is released before ADP, and that the inhibitor has arrested the catalytic cycle of ATP hydrolysis immediately preceding the release of the nucleotide. Subsequent release of the nucleotide would provide the β_E_-state observed in the ‘ground state’ structures of F_1_-ATPase where no nucleotide is bound to this subunit. One cautionary note is that it is possible that the position of α-helix C3 in the C-terminal domain of the β_E_-subunit, carrying residue β_E_-F424, could be influenced by a contact in the crystal lattice with α-helix b (residues 102–110) in the δ-subunit of an adjacent F_1_-complex (figure 7). As residues β_E_-F424A and β_E_-Y345 provide the pocket for binding the adenosine moiety of ADP molecule, one possible interpretation is that α-helix C3 and the adenosine binding pocket are being held artificially in this position by the crystal contact. However, one significant argument against this interpretation is that a closely related conformation of a β_E_-subunit, containing a bound ADP molecule, also lacking an associated magnesium ion, has been observed independently in a structure of bovine F_1_-ATPase, known as F_1_-PH, crystallized in the presence of nucleotides, magnesium ions and phosphonate, a chelating agent for magnesium [11]. Global superimposition of the β_E_-subunits in the yF_1_-I1–53 and F_1_-PH structures demonstrates that the conformations and nucleotide occupancies of the nucleotide binding sites are essentially identical (r.m.s. value 0.79 Å; figure 7), and that there are no similar crystal contacts that could influence the conformation of the β_E_-subunit in F_1_-PH. Thus, both structures appear to represent a post-hydrolysis pre-nucleotide release step in the catalytic cycle of the enzyme.

Alternatively, it can be argued that the presence of the bound inhibitor protein distorts the structure of F_1_-ATPase and leads to an inhibited state that is not on the active pathway of ATP hydrolysis. The close similarity of the β_E_-subunit in the present and bovine F_1_-PH structures (figure 7) can be taken as evidence against this interpretation. There is no inhibitor protein bound to F_1_-PH, and the structure of the β_E_-subunit fits well with the interpretation that it represents a post-hydrolysis, pre-nucleotide release state in the catalytic cycle. The other structural changes in yeast F_1_-ATPase, described earlier, associated with the presence of the bound inhibitor are quite minor, as a global superimposition of the current structure with the yeast ground state structure demonstrates. Using ‘complex I’ from the yeast ground state structure, the r.m.s values are 1.27 Å for the whole complexes and 1.02 Å for the α_3_β_3_-domains. Therefore, the preferred interpretation is that the structure represents an intermediate in the catalytic cycle.

At present, there is no clear explanation of why the bovine and yeast inhibitors arrest the catalytic cycle of their cognate F_1_-ATPases at different points. Structures of the bovine enzyme inhibited with the yeast inhibitor and vice versa might help to elucidate this point. However, the observation does raise the prospect that it may be possible to engineer inhibitor proteins to arrest the catalytic cycle of the active enzyme at other points and thereby capture other states in the catalytic cycle for structural analysis.

### 4.2. Mechanism of hydrolysis of ATP by F_1_-ATPase

One important conclusion reached from pairwise comparisons of all the known structures of F_1_-ATPase (with the possible present exception of yeast F_1_-I1–53) is that the conformations of the catalytic sites in the three β-subunits of the enzyme are not influenced by contacts between neighbouring complexes in the lattice of the crystals used to determine the structures [14], contrary to what has been proposed [15–19]. Therefore, the structures of the catalytic sites in a ‘ground state’ structure [20,21] and in a ‘transition state’ analogue structure [22] define steps in the catalytic pathway of ATP hydrolysis by F_1_-ATPase, as described previously and summarized in the electronic supplementary material, figure S3. In the ‘ground state’ structure, the three catalytic sites in the β-subunits of the enzyme have different structures that are imposed by the asymmetry of the γ-subunit in the central stalk of the enzyme. They are usually referred to as the β_DP_-, β_TP_- and β_E_-subunits. In a catalytic cycle, each catalytic subunit passes through each of these states, and the conversion of one state to another is brought about by the rotation in 120° steps of the central stalk in an anticlockwise fashion (as viewed from the membrane domain of the intact F-ATPase). Each 360° rotation is accompanied by the hydrolysis of three ATP molecules. In the ground state structure, the β_E_-subunit has no nucleotide bound to its nucleotide binding site. A 120° rotary step converts the β_E_-subunit to the β_TP_-subunit, entrapping an ATP molecule in the nucleotide binding site. The next 120° step converts the β_TP_-subunit to the β_DP_-subunit, poising the site for ATP hydrolysis. In the next 120° step hydrolysis occurs; the products magnesium.ADP and phosphate are released from the enzyme and the β_E_-subunit is regenerated. In the transition state analogue complex, the conformation of the β_E_-subunit is intermediate between those of the β_DP_- and β_E_-subunits, and it defines the state of the enzyme during the cleavage of the γ-phosphate from ATP by nucleophilic attack by a water molecule, itself activated by β_DP_-E189 (β_DP_-E188 in the bovine enzyme). The present structure defines the conformation of the β_E_-subunit after the β_E_-subunit in the transition state analogue structure, and before the formation of that of the β_E_-subunit in the ground state structure. In this state, the magnesium ion and phosphate have been released, but the nucleotide, ADP, is still bound to the β_E_-subunit. It is incorporated into the pathway of ATP hydrolysis (see electronic supplementary material, figure S3). In two other NTPases, protein-1A (a member of the kinesin superfamily) [23] and *ras*p21 [24], it has been shown similarly that following the hydrolysis of an NTP molecule, the magnesium ion is released before the product NDP.

The evidence concerning the order of release of the magnesium ion and phosphate from F_1_-ATPase is at first sight somewhat contradictory. In one of the three copies of the enzyme in the ‘ground state’ crystal structure of yeast F_1_-ATPase, phosphate (or sulphate) remains bound in the β_E_-subunit in a position approximately 8 Å from the γ-phosphate of AMP–PNP in other nucleotide binding sites, suggesting that it is released last [25]. However, there is no phosphate bound in the other two copies of the enzyme in the crystal lattice, or in the current structure, suggesting that in the yeast enzyme, both the magnesium ion and phosphate are released before the nucleotide in an unknown order. In the bovine enzyme, the evidence also suggests that both magnesium and phosphate are released before the nucleotide. Although there is often density in ‘ground state’ structures of bovine F_1_-ATPase close to the P-loop that can be interpreted as a bound anion, when this density is modelled as a phosphate group the temperature factors are very high, and there are only a few favourable interactions with the protein. In addition, this feature lies in a position midway between the α- and β-phosphates of a bound nucleotide, and therefore could not be occupied at the same time. Also, it is remote (7–8 Å) from the clearly identified phosphate binding site in the ‘half-closed’ β_E_-subunit of the bovine transition state complex [22] and in the β_E_-subunit of the yeast ‘ground state’ structure [25]. Therefore, probably this site is an anion binding pocket that is occupied fortuitously by phosphate or sulphate in the crystal structures, and it does not represent a catalytically or physiologically relevant phosphate binding site. In addition, in the structure of bovine F_1_-PH where nucleotide is bound to the β_E_-subunit, there is no bound phosphate or magnesium ion, suggesting that they are released before the nucleotide.

## 5. Material and methods

### 5.1. Analytical methods

Protein concentrations were estimated by the bicinchoninic acid assay (Pierce, Thermo Scientific, Rockford, IL, USA). Purified proteins were analysed by SDS–PAGE [26] and stained with Coomassie blue dye. ATPase activity was measured as described before [1]. Molecular masses of proteins were measured following electrospray ionization in a Quattro Ultima triple quadrupole mass spectrometer (Waters, Milford, MA, USA).

### 5.2. Protein overexpression

A fragment of bovine IF_1_ from residues 14 to 60 fused to glutathione-*S*-transferase (GST) with a C-terminal His_6_ tag (known as bIF_1_14–60-GST-His_6_) was over-expressed as described before [10]. The sequence encoding residues 1–53 of IF_1_ from *S. cerevisiae* (yI1–53) was amplified by PCR from a pRUN vector containing the coding sequence for yeast IF_1_ containing the mutation E21A. This mutation abolishes the pH sensitivity of the inhibitory action of the inhibitor protein [13], and therefore it offers the prospect of conducting crystallization experiments involving the inhibited complex over a wider range of pH values. The forward and reverse primers, respectively, were 5′-TAATACGACTCACTATAGGG-3′ and 5′-CAGAAGCTTTTAAGAATCAATCTTCTTTCGTTG-3′. The product was digested with *Nde*I and *Hin*dIII, and cloned into the pRun plasmid. yI1–53 was over-expressed for 12 h at 25°C in *Escherichia coli* strain BL21(DE3), as described previously [27].

The sequence encoding residues 1–53 of IF_1_ from *S. cerevisiae* (yI1–53) together with a hexahistidine tag was amplified by PCR from the yI1–53 pRun plasmid. Single point amino acid substitutions were introduced into this sequence with a series of pairs of synthetic complementary oligonucleotide primers containing the mutated codons and 24 bases 5′ and 3′ of the codon, respectively. This region was amplified by PCR, and extended in a second PCR with primers flanking the 5′ and 3′ ends of the coding sequence for yI1–53His. The modified sequences were cloned into the pRun vector. The mutant proteins plasmids were over-expressed from these expression plasmids for 4 h at 25°C in *E. coli* strain C41(DE3) [28].

### 5.3. Protein purification

The full-length bovine and yeast inhibitor proteins and bIF_1_14–60-GST-His_6_ were expressed and purified as described previously [10]. The C-terminally truncated yeast inhibitor protein yI1–53 containing the mutation E21A and lacking a His-tag was used in crystallization experiments. It was expressed as described before and purified at 4°C as follows. Cells containing yI1–53 were suspended in buffer containing 50 mM Tris–HCl, pH 7.4, 100 mM NaCl, 25 mM imidazole, 5 mM benzamidine hydrochloride, 5 mM 6-aminocaproic acid, 0.005 per cent phenylmethylsulfonyl fluoride, 0.02 per cent sodium azide and one tablet per 50 ml of an EDTA-free protease inhibitor mixture (Roche Diagnostics, Mannheim, Germany). The cells were disrupted by two passages at 30 000 psi through a Z Plus 2.2 kW cell disruptor (Constant Systems, Daventry, UK). The broken cells were centrifuged (200 000*g*, 90 min), and the supernatant was filtered through a membrane (0.22 μm pore size, Sartorius, Göttingen, Germany). The filtrate was heated to 60°C under nitrogen for 20 min in the presence of 5 mM dithiothreitol, centrifuged (9000*g*, 15 min) and dialysed for 12 h against a buffer containing 20 mM 1,3-diaminopropane, pH 10.5, 1 mM EDTA, 1 mM benzamidine hydrochloride, 1 mM 6-aminocaproic acid, 0.005 per cent phenylmethylsulfonyl fluoride and 0.02 per cent sodium azide. It was loaded onto a Hi-Trap Q column (5 ml; GE Healthcare Bio-Sciences AB, Uppsala, Sweden). Pure yI1–53 was recovered in the breakthrough fractions. It was dialysed for 10 h against 20 mM Tris–HCl, pH 7.4, concentrated to 10 mg ml^−1^ and stored at −25°C. Cells containing mutant yI1–53His proteins were broken as described earlier. They were applied to a Hi-Trap nickel Sepharose column (GE Healthcare, Buckinghamshire, UK) equilibrated in buffer containing 20 mM Tris–HCl, pH 7.4, 10% (v/v) glycerol, 25 mM imidazole and 0.1 M sodium chloride. yI1–53His and mutant forms were eluted with a linear gradient of imidazole from 25 to 300 mM in a total volume of 100 ml. Fractions containing the proteins were pooled and dialysed for 4 h against 2 l of buffer consisting of 20 mM Tris–HCl, pH 7.4, and concentrated with a VivaSpin concentrator (molecular weight cut-off 5 kDa; Sartorius, Göttingen, Germany). The analysis of the purified inhibitors by SDS–PAGE and their mass spectrometric characterization are shown in electronic supplementary material, figure S2 and table S1, respectively.

### 5.4. Purification of F_1_-ATPase

*Saccharomyces cerevisiae* (strain W303-1A; kindly provided by B.L. Trumpower, Dartmouth Medical School, NH, USA) was grown at 30°C in an Applikon ADI 1075 fermenter (Applikon, Schiedam, Netherlands) in 55 l of medium consisting of 1 per cent yeast extract, 2 per cent peptone, 3% v/v glycerol and adenine (0.055 g l^−1^), pH 5.0. When the culture had reached late exponential phase (*A*_600_ 8.0–9.0), the cells were cooled to 18°C and harvested at 18 000*g* in a continuous flow centrifuge. The following procedures were carried out at 4°C. Yeast cells (1.8 kg) were suspended in 2 vol (v/w) of buffer consisting of 100 mM Tris–HCl, pH 8.0, 650 mM sorbitol, 5 mM EDTA, 5 mM benzamidine, 5 mM 6-aminohexanoic acid, 0.005 per cent phenylmethylsulfonyl fluoride and 0.2 per cent bovine serum albumin. The suspension was passed through a Dyno-Mill bead mill (W. A. Bachofen Machinery, Basel, Switzerland). The pH of the broken cells was adjusted to pH 8.0 with Trizma (3 M). Debris was removed by centrifugation at 7500*g* for 30 min. Mitochondria were recovered from the supernatant by centrifugation at 26 000*g* for 45 min. They were washed twice with buffer containing 20 mM Tris–HCl, pH 7.5, 650 mM sorbitol, 1 mM EDTA, 5 mM benzamidine, 5 mM 6-aminohexanoic acid and 0.005 per cent phenylmethylsulfonyl fluoride, and re-suspended in buffer containing 50 mM Tris–HCl, pH 8.0, 250 mM sucrose, 5 mM benzamidine hydrochloride, 5 mM 6-aminocaproic acid and one tablet per 50 ml of an EDTA-free protease inhibitor mixture. Submitochondrial particles were prepared as described previously [29]. F_1_-ATPase from a 50 ml portion of submitochondrial particles (20 mg ml^−1^) was released with chloroform [29] in the presence of 2 mM ADP and 4 mM magnesium sulphate. A tablet of an EDTA-free protease inhibitor cocktail, a solution (250 μl) containing 1 M of each of benzamidine hydrochloride and 6-aminohexanoic acid, and of mixture (250 μl in methanol) of 10 mM each of amastatin, besatatin, pepstatin, leupeptin and diprotin were added. A twofold molar excess of bIF_1_14–60-GST-His_6_ and 100 μl of a neutralized stock solution containing 500 mM ATP and 1 M magnesium sulphate were added at 23°C. Two further portions of 100 μl of the same solution were added after 5 and 10 min. Methanol (10%), sodium chloride (150 mM) and dithiothreitol (5 mM) were added to the solution of the inhibited complex. It was applied at 4°C with a flow rate of 0.5 ml min^−1^ to a GSTrap affinity column (5 ml; GE Healthcare Bio-Sciences AB) equilibrated in buffer consisting of 50 mM Tris–HCl, pH 8.0, 10 per cent methanol, 250 mM sucrose, 150 mM sodium chloride, 1 mM ATP, 2 mM magnesium sulphate, 5 mM benzamidine hydrochloride, 5 mM 6-aminocaproic acid, 5 mM dithiothreitol and one tablet per 50 ml of an EDTA-free protease inhibitor mixture. The column was washed with the same buffer, then transferred to 23°C and then washed again with the same buffer containing 50 mM EDTA and 50 mM EGTA, and no methanol or DTT, and with ADP instead of ATP, to release the active F_1_-ATPase. The recovered yeast F_1_-ATPase was passed through a column of Superdex 200 (10/300; GE Healthcare Bio-Sciences AB) at 0.5 ml min^−1^. The specific activity of the purified enzyme was 216 U mg^−1^. The analysis of the purified enzyme by SDS–PAGE and the mass spectrometric characterization of its subunits are shown in the electronic supplementary material, figure S2 and table S1, respectively.

### 5.5. Molecular mass estimation of inhibited complexes

All procedures were performed at 23°C. F_1_-ATPase (1 mg ml^−1^; 100 μl) from *S. cerevisiae* in F_1_ buffer consisting of 50 mM 3-morpholinopropanesulfonic acid, pH 6.6, 10% (w : v) glycerol, 1 mM ADP, 2 mM magnesium sulphate and 0.002% (w/v) phenylmethanesulfonylfluoride was mixed with a 15-fold molar excess of IF_1_ from *S. cerevisiae* (10 mg ml^−1^; 3 μl) or a fivefold molar excess of bovine IF_1_ (10 mg ml^−1^; 1.3 μl) together with 1 mM ATP and 2 mM magnesium sulphate (2 μl from a stock solution containing 20 mM ATP and 40 mM magnesium sulphate). Further portions (2 μl) of the solution of ATP and magnesium sulphate were added after 5 min and 10 min. The inhibited complexes and the active F_1_-ATPase were applied separately to a column of Superose 6 (10/300, GE Healthcare Bio-Sciences AB) pre-equilibrated with F_1_ buffer. The absorbance of the eluate was monitored at 280 nm, and fractions were analysed by SDS–PAGE. The void volume of the column was determined with Blue Dextran 2000. The column was calibrated with thyroglobulin, catalase and the monomeric and dimeric forms of ferritin.

### 5.6. Assay of inhibition of F_1_-ATPase

The ATP hydrolase activity of yF_1_-ATPase in the presence of the various mutant inhibitors was measured with an ATP-generating system as described before [1] by addition of 2.5 μg of F_1_-ATPase (specific activity; 101 μmol min^−1^ mg^−1^) to 1 ml of assay mixture at 37°C. The absorbance at 340 nm was recorded for 10 min with each inhibitor at six different concentrations.

The rate constants of binding to and dissociation from F_1_-ATPase, *k*_on_ and *k*_off_, respectively, of each inhibitor protein were measured from the exponential decay of the rate of ATPase activity after addition of various amounts of inhibitor protein as described previously [10]. The dissociation constant, *K*_i_, for the binding of the inhibitor to the enzyme was calculated from *K*_i_ = *k*_off_/*k*_on_.

### 5.7. Crystallization of the yeast F_1_-I1–53 complex

Active F_1_-ATPase (12 mg ml^−1^) was exchanged on a Biospin-6 desalting column (BioRad, Hemel Hempstead, UK) into crystallization buffer, prepared in D_2_O and consisting of 100 mM Bis–Tris propane, pH 7.5, 100 mM sucrose, 1 mM ADP and 10 mM magnesium sulphate. Then, the enzyme was inhibited at 23°C with a fourfold molar excess of yI1–53 (containing the mutation E21A and lacking a His-tag) in the presence of 1 mM ATP and 2 mM magnesium sulphate. Further portions (5 μl of a neutralized stock solution containing 200 mM ATP and 400 mM magnesium sulphate ml^−1^ protein solution) were added after 5 and 10 min. More than 95 per cent of the ATP hydrolysis activity of the enzyme was inhibited. Sodium–potassium tartrate was added to 100 mM, and the concentration of the protein solution was adjusted to 10 mg ml^−1^ with crystallization buffer. Crystals were grown at 23°C in 72 well micro-batch plates (Nunc International, Thermo Fisher Scientific, Roskilde, Denmark) under filtered paraffin oil (BDH laboratory supplies, Poole, UK). The crystallization drops (4 μl) contained a 1 : 1 mixture of protein solution and precipitant solution (20–26% polyethylene glycol 3000 and 600 mM NaCl prepared in D_2_O). Crystals appeared after 2 days and were fully grown after two weeks. They were harvested into a solution identical to the crystallization drop, but containing an additional 1% (w/v) polyethylene glycol 3000. They were cryoprotected with 20% (v/v) ethylene glycol introduced in 5 per cent steps with 3 min at each step. The cryoprotected crystals were harvested with Micro-Mount cryoloops (MiTeGen, Ithaca, NY, USA), plunge-frozen in liquid nitrogen and stored at 100 K.

### 5.8. Data collection

Diffraction data were collected at 100 K at 2.5 Å resolution using a MX300 charge-coupled detector (Rayonix, Evanston, IL, USA) on beamline I24 (*λ* = 0.977 Å) at the Diamond Light Source, Harwell, UK. The data were processed with iMOSFLM [30] and with programs POINTLESS, SCALA [31] and CTRUNCATE [32] from the Collaborative Computational Project Number 4 (CCP4) suite [33].

### 5.9. Solution and refinement of the structure

The structure of yF_1_-I1–53 from *S. cerevisiae* was solved by molecular replacement with PHASER [34]. The starting model was complex I taken from a ground state structure of F_1_-ATPase from *S. cerevisiae* (protein data bank 2HLD) [25]. Nucleotides, magnesium ions and water molecules were removed from the model. Rigid body refinement, restrained refinement and non-crystallographic symmetry refinement were performed with REFMAC5 [35]. Manual rebuilding and the addition of water molecules were performed with COOT [36], alternating with refinement performed with REFMAC5. For calculations of the *R*_free_ value, 5 per cent of the diffraction data were excluded from the refinement. Stereochemistry was assessed with MolProbity [37] and figures were prepared with PyMol [38]. The structure was compared with other structures using the SUPER alignment tool in PyMol with refinement cycles set to zero. Coordinates and structure factors for the described structure have been deposited with the protein data bank under the accession code 3zia.

## 6. Acknowledgements

J.E.W. designed and supervised the project. G.C.R., J.V.B., M.G.M. and I.M.F. carried out the experiments. J.E.W., M.G.M., A.G.W.L. and G.C.R. analysed the data. J.E.W., G.C.R., M.G.M. A.G.W.L. and D.M.M. prepared the manuscript. We thank S. Palmer for growing cells of *S. cerevisiae*, D. M. Rees for a sample of pure bIF_1_14–60-GST-His_6_, and the staff at beamline I24 at the DIAMOND Light Source for help with collection of data. Support for this work was provided by the Medical Research Council, UK, including a PhD studentship (to G.C.R.) and a Career Training Fellowship (to J.V.B.), by the European Drug Initiative in Channels and Transporters (EDICT; to J.E.W.), and by a grant from NIH no. R01GM66223 to D.M.M. The authors declare that they have no conflict of interest.

## Supplementary Material

Supplementary information for The structure of F1-ATPase from Saccharomyces cerevisiae inhibited by its regulatory protein IF1
