# Supplementary material for: The structure of F1-ATPase from Saccharomyces cerevisiae inhibited by its regulatory protein IF1
Source: Open Biol. 2013 Feb;3(2):120164. doi: 10.1098/rsob.120164 (PMC3603450; doi:10.1098/rsob.120164)
Supplement: Supplementary information for The structure of F1-ATPase from Saccharomyces cerevisiae inhibited by its regulatory protein IF1 [file rsob120164-s1.pdf]

Supplementary information for

The structure of F_1-ATPase from *Saccharomyces cerevisiae* inhibited by its regulatory protein IF_1

Graham C. Robinson[1], John V. Bason[1], Martin G. Montgomery[1], Ian M. Fearnley[1], David M. Mueller[2], Andrew G. W. Leslie[3] and John E. Walker[1]

[1]The Medical Research Council Mitochondrial Biology Unit, Hills Road, Cambridge, CB2 0XY, United Kingdom; [2]Rosalind Franklin University of Medicine and Science, The Chicago Medical School, North Chicago, IL, 60064, USA; [3]The Medical Research Council Laboratory of Molecular Biology, Hills Road, Cambridge, CB2 2QH, United Kingdom

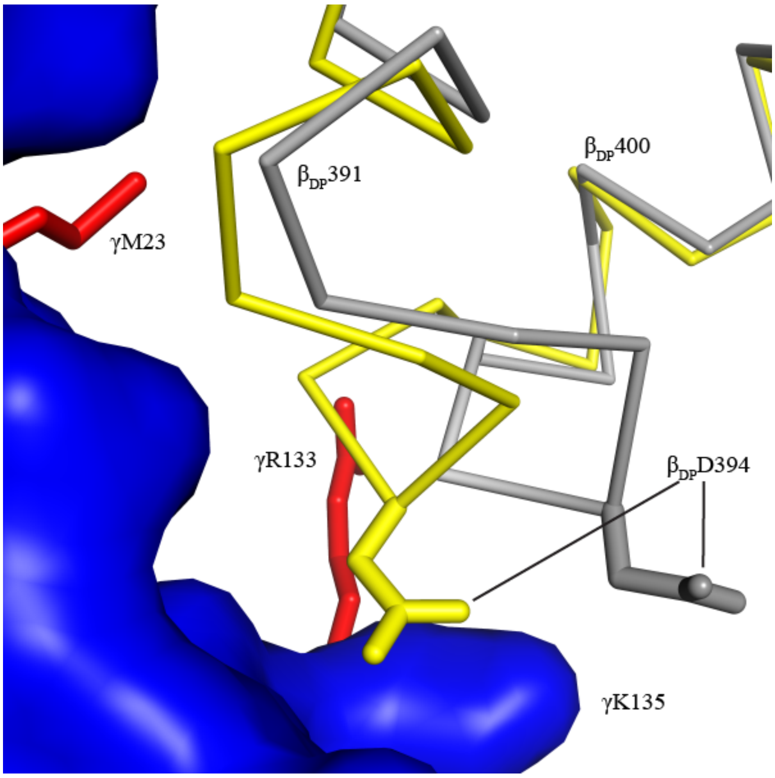

Fig S1. A local difference in the structures of bovine F_1-I1-60 and yF_1-I1-53 that accommodates a sequence difference in the yeast and bovine inhibitor proteins. The Figure illustrates the different conformations adopted by residue β_{DP}-D394 in the loop consisting of residues 391-398; the yeast and bovine loops are depicted in yellow and grey stick representation, respectively. Relevant side-chains of the bovine γ-subunit are shown in red, and then surface representation of the yeast γ-subunit is blue. The conformation of the loop containing β_{DP}-D394 in the bovine enzyme is influenced by γR133 and γM23 (both red), placing β_{DP}-D394 in a position where it interacts with residue R32 (not shown) in bovine IF_1. In yeast IF_1, R32 is replaced by F27 (not shown) forcing β_{DP}-D394 to adopt a different conformation, which is accommodated by a change in the position of the side-chain of yeast γK135 (blue solid representation), the equivalent of bovine γR133.

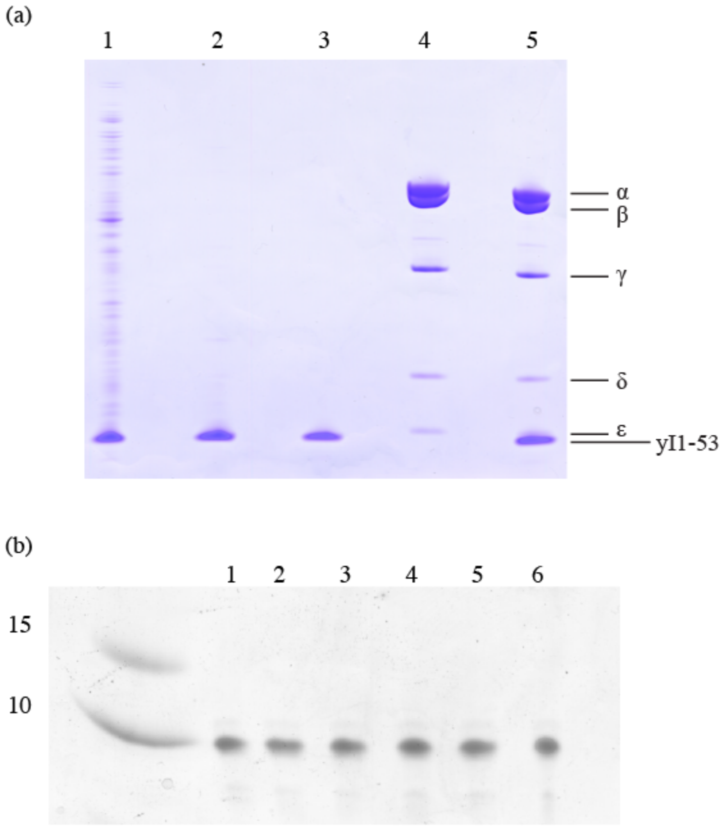

Fig. S2. Analysis of proteins. The proteins were analyzed by SDS-PAGE and stained with Coomassie blue dye. Part (a); lane 1, supernatant following breakage of cells of *E. coli* BL21(DE3) expressing the yeast inhibitor protein, I1-53; lanes 2 and 3, samples before and after application to Hi-Trap Q-column, respectively; lane 4, purified yeast F_1-ATPase; lane 5, the yeast F_1-I1-53 inhibited complex. Part (b); characterization of mutant proteins of the yeast inhibitor yI1-53His. The proteins contain the following mutations: lane 1, E2A; lane 2, R9A; lane 3, D15A; lane 4, R30A; lane 5, L37A; lane 6, L40A. Molecular weight markers are shown on the left.

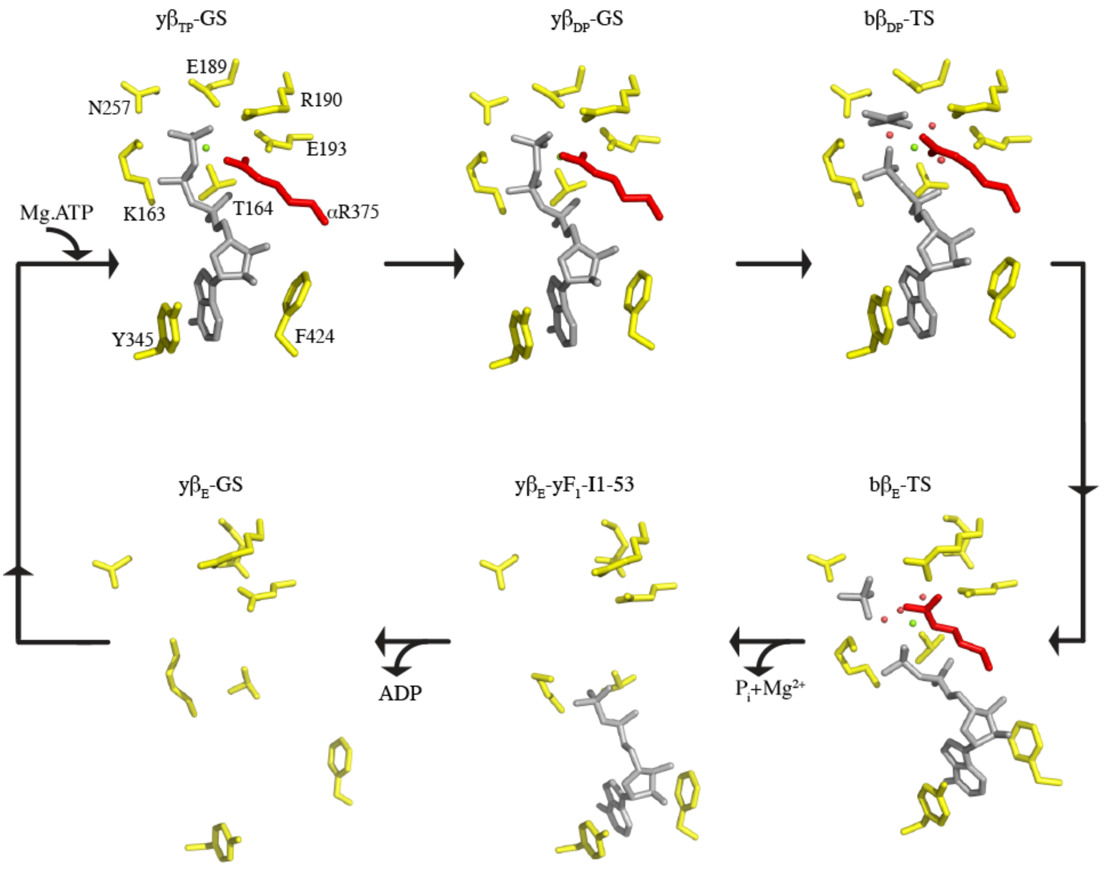

Fig. S3. The structural basis of the hydrolysis of ATP by F_1-ATPase. In the upper and lower rows the conformations of the various subunits are taken from the yeast (y) ground-state (GS) and bovine (b) transition state analog (TS) structures and from the β_E-subunit of the current structure. The β_E-, β_{TP}- and β_{DP}-subunits are placed in the standard order described before [18], conversion from one state to the next requiring a 120° rotary step of the central stalk of the enzyme. In the conversion β_EGS to β_{TP}GS, magnesium.ATP is bound to the catalytic subunit. The next 120° rotation converts β_{TP}GS to β_{DP}GS, the catalytically active site of the enzyme. In the next 90° rotary sub-step (probably), the transition state forms; at this point in the Figure, the bovine subunit, bβ_{DP}-TS, is depicted. In the next 30° rotary sub-step (probably), scission of the γ-phosphate of ATP occurs (depicted as the bovine subunit, bβ_E-TS), and is followed by the release of the magnesium ion and phosphate, producing the state of the β_E-subunit in the current structure. Finally, the nucleotide is released, regenerating

yβ_E-GS. The magnesium ion and the nucleotide are shown in green and grey respectively, and arginine finger residue αR375 is red. In the bovine bβ_{DP}-TS and bβ_E-TS structures the red spheres represent water molecules involved in coordination of the magnesium ion. In bβ_{DP}-TS and bβ_E-TS, the AlF_4^- and phosphate moieties are depicted in grey.

Table S1. Molecular masses of subunits of F_1-ATPase from *S. cerevisiae* and of yeast inhibitor proteins.

Protein	Mass (Da)		Mass difference	Modification
	Observed	Calculated		
α	54950.6	54944.7	+ 5.9	None
β	51131.6	51126.4	+ 5.2	None
γ	30618.4	30616.2	+ 2.2	None
δ	14554.1	14553.5	+ 0.6	None
ε	6611.6	6611.4	+ 0.2	None
I1-63	7383.6	7383.2	+ 0.4	None
I1-53	6165.2	6164.8	+ 0.4	None
I1-53His	6987.8	6987.7	+ 0.1	None
E2A I1-53His	6930.0	6929.6	+ 0.4	None
R9A I1-53His	6902.4	6902.5	- 0.1	None
D15A I1-53His	6944.0	6943.6	+ 0.4	None
R30A I1-53His	6902.5	6902.5	0	None
L37A I1-53His	6946.1	6945.6	+ 0.5	None
L40A I1-53His	6946.1	6945.6	+ 0.5	None

Subunits of F_1-ATPase were separated by reverse phase chromatography, and their masses were measured “on-line” by ESI-MS. Calculated values are based on the mature protein sequences. All of the yeast inhibitor proteins contain the mutation E21A.
